# Integrated, Community-Led, Culturally Safe Implementation of a Chronic Kidney Disease Detection Program for Indigenous Communities of Canada: The Kidney Check Program Report

**DOI:** 10.1177/20543581261447194

**Published:** 2026-05-30

**Authors:** Somkanya Tungsanga, Cathy Woods, Selina Allu, Allison B. Dart, Arsh K. Jain, Lene Jorgensen, Michelle Hampson, Oksana Harasemiw, Marc Hébert, Joanne Kappel, Nicki Kirlin, Carmen Levandoski, Meg Lunney, Cynthia MacDonald, Ikechi G. Okpechi, Leta Philp, Stacey Shmyr, Catherine Turner, David Collister, Harley Crowshoe, Heather Harris, Erin Hedin, Jocelyn Jones, Maureena Loth, Paul Komenda, Adeera Levin, Aminu K. Bello

**Affiliations:** 1 Division of Nephrology & Immunology, Department of Medicine, Faculty of Medicine & Dentistry, University of Alberta, Edmonton, Canada; 2 Can-SOLVE CKD Network, Vancouver, BC, Canada; 3 University of Calgary – Medicine, AB, Canada; 4 Children’s Hospital Research Institute of Manitoba, University of Manitoba, Winnipeg, Canada; 5 Division of Nephrology, Department of Medicine, Western University, and London Health Sciences Centre, ON, Canada; 6 Alberta Health Services/Acute Care Alberta, Calgary, Canada; 7 Chronic Disease Innovation Centre, Seven Oaks General Hospital, Winnipeg, MB, Canada; 8 Ontario Health (Ontario Renal Network), Toronto, Canada; 9 College of Medicine, University of Saskatchewan, Saskatoon, Canada; 10 Saskatchewan Health Authority, Saskatoon, Canada; 11 Department of Medicine, University of Calgary, AB, Canada; 12 Section of Nephrology, Department of Internal Medicine, University of Manitoba, Winnipeg, Canada; 13 Division of Nephrology, Department of Pediatrics, Faculty of Medicine & Dentistry, University of Alberta, Edmonton, Canada; 14 Division of Nephrology, Department of Medicine, University of British Columbia, Vancouver, Canada

**Keywords:** CKD detection, Indigenous peoples, community-based, implementation science, health equity, détection de l’IRC, Premières Nations, approche communautaire, science de la mise en œuvre, équité en santé

## Abstract

**Purpose of program:**

Indigenous peoples in Canada experience a disproportionate burden of chronic kidney disease (CKD) yet face systemic, geographic, and cultural barriers to early detection and care. The Kidney Check program was established as a national initiative to address this gap by providing community-led, culturally safe, and integrated chronic disease screening.

**Sources of information:**

Kidney Check builds on the success of the First Nations Community-Based Screening to Improve Kidney Health and Prevent Dialysis (FINISHED) study in Manitoba and was expanded through the Can-SOLVE CKD Network. This report summarizes updated data from April 2023 to March 2024, capturing the implementation of Kidney Check across five provinces: Alberta (AB), British Columbia (BC), Manitoba (MB), Ontario (ON), and Saskatchewan (SK). Data sources include provincial program records, training logs, process measures, referral data, patient and provider testimonials, and qualitative evaluations from community members, providers, and patient partners.

**Methods:**

Kidney Check applies a standardized, mobile, point-of-care-testing (POCT)-based screening protocol for CKD, diabetes, and hypertension, adapted locally in collaboration with Indigenous communities. Implementation followed principles of Indigenous governance, data sovereignty (Ownership, Control, Access, Possession [OCAP]), and Two-Eyed Seeing, where both Western and Indigenous medicines and ways are acknowledged, respected, and practiced, with provincial adaptations tailored to local contexts. Information was synthesized across jurisdictions to identify process measures, barriers and facilitators to implementation, and early outcomes.

**Key findings:**

Since program inception, thousands of Indigenous participants have been screened across more than 50 communities. AB and BC demonstrated scale-up through train-the-trainer and community-embedded models, training over 30 providers and screening >700 participants in BC alone. Manitoba built on the FINISHED study to deliver screening in 10 communities, with nearly 700 participants screened. Ontario resumed screening in 2024, with >250 participants screened across five regional renal programs. Saskatchewan launched Kidney Check in 2023, screening 230 participants, with early identification of intermediate- and high-risk individuals. Across sites, facilitators included strong community partnerships, local hiring, and integration with existing health services. Barriers included logistical challenges (equipment, travel, quality assurance and quality control [QA/QC]), staffing constraints, and sustainability concerns.

**Limitations:**

Variation in infrastructure, referral pathways, and data systems across provinces limits cross-jurisdictional comparability. Long-term outcome data (eg, CKD progression, cardiovascular events, mortality) are not yet available.

**Implications:**

The Kidney Check program demonstrates that culturally safe, community-led, POCT-based screening for CKD and related chronic diseases is feasible, acceptable, and scalable across diverse Indigenous communities in Canada. Lessons learned highlight the central importance of Indigenous leadership, cultural safety, and flexible delivery models to advance health equity.

## Abrégé

**Objectif du programme:**

Au Canada, les populations autochtones sont disproportionnellement touchées par l’insuffisance rénale chronique (IRC) et se heurtent à des obstacles systémiques, géographiques et culturels qui limitent la détection précoce et l’accès aux soins. Le programme Kidney Check, une initiative nationale, vise à combler cette lacune par un dépistage intégré, culturellement sécuritaire et piloté par les communautés.

**Sources de l’information:**

Kidney Check s’appuie sur les résultats de l’étude FINISHED (First Nations Community Based Screening to Improve Kidney Health and Prevent Dialysis) menée au Manitoba, a été élargi par l’entremise du réseau CAN-SOLVE CKD. Ce rapport résume les données d’avril 2023 à mars 2024 sur son déploiement dans cinq provinces (Alberta, Colombie-Britannique, Manitoba, Ontario et Saskatchewan) provenant de registres provinciaux, données de formation, indicateurs de processus, données de référence, ainsi que des témoignages et évaluations qualitatives des membres des communautés, des prestataires de soins et des partenaires patients.

**Méthodologie:**

Kidney Check utilise un protocole mobile et standardisé de dépistage par analyses hors laboratoire (AHL) pour l’IRC, le diabète et l’hypertension, adapté avec les communautés autochtones. La mise en œuvre a été guidée par les principes de gouvernance des données des Premières Nations (PCAP) et de la vision « Two-Eyed Seeing », qui vise à reconnaître, respecter et combiner les approches médicales et les savoirs occidentaux et autochtones, avec des adaptations provinciales propres aux contextes locaux. Les informations ont été analysées par provinces, afin d’identifier les indicateurs de processus, les obstacles, les facilitateurs et les résultats précoces.

**Principales observations:**

Depuis sa mise en œuvre, Kidney Check a recruté des milliers de participants des Premières Nations dans plus de 50 communautés. En Alberta et en Colombie-Britannique, des modèles de formation en cascade (« train-the-trainer ») et d’intégration communautaire ont permis de former plus de 30 prestataires et de dépister plus de 700 personnes en Colombie-Britannique seulement. Au Manitoba, le dépistage fondé sur l’étude FINISHED a rejoint dix communautés (près de 700 personnes). En Ontario, le programme a repris en 2024 (plus de 250 personnes dans cinq programmes régionaux), tandis qu’en Saskatchewan, le programme lancé en 2023 a permis de dépister 230 personnes et d’identifier rapidement les individus présentant un risque intermédiaire ou élevé. Dans l’ensemble des sites, les facilitateurs incluaient des partenariats communautaires robustes, le recrutement local et l’intégration aux services de santé existants. Les obstacles comprenaient des défis logistiques (équipement, déplacements, AQ/CQ), des contraintes liées aux effectifs et des enjeux de pérennité.

**Limites:**

Les disparités en matière d’infrastructures, de circuits d’aiguillage et de systèmes de données entre les provinces restreignent la comparabilité entre juridictions. Les données sur les résultats à long terme (progression de l’IRC, événements cardiovasculaires, mortalité) ne sont pas encore disponibles.

**Conclusion:**

Le programme Kidney Check démontre la faisabilité, l’acceptabilité et la capacité de déploiement à grande échelle d’un dépistage des maladies chroniques, incluant l’IRC, par analyses hors laboratoire (AHL), piloté par les communautés et culturellement sécuritaire, dans diverses communautés des Premières Nations au Canada. Le programme met en évidence l’importance du leadership autochtone, de la sécurité culturelle et de modèles de prestation flexibles pour l’amélioration de l’équité en santé.

## Purpose of Program

Chronic kidney disease (CKD) is often underdiagnosed and undertreated in Indigenous populations across Canada, particularly in rural and remote regions.^1^ Despite a disproportionate burden of disease,^2-4^ systemic, geographic, and cultural barriers continue to limit access to early detection and timely follow-up care.^5-7^ This evidence-practice gap contributes to delayed diagnoses, avoidable complications, and poor health outcomes.^8^

The Kidney Check program was established to address this gap through an integrated, community-led, and culturally safe screening initiative.^9,10^ Using portable point-of-care testing (POCT), the program screens for major non-communicable diseases (NCDs), including CKD, diabetes, hypertension, and obesity within Indigenous communities across Canada. This model combines the traditional, multi-step screening process into a single visit where participants receive services directly where people live, overcoming many access barriers. The program is co-designed with Indigenous communities, Elders/Knowledge Keepers, health care providers, and patient partners to ensure it reflects community priorities and traditional values. The communities involved, which are primarily First Nations but have also included some Métis communities, appreciate the immediate access to test results, personalized health coaching, and the opportunity to engage in care that is respectful, locally delivered, and culturally safe.^11^ The Kidney Check program aims to promote early identification of chronic disease while empowering communities through respectful partnership and capacity building. It demonstrates how Indigenous-led innovation can advance health equity and improve kidney and overall health outcomes across Canada.

### From FINISHED to the Can-SOLVE CKD Network

The Kidney Check program was conceived as a national initiative to operationalize the priorities outlined in the Canadians Seeking Solutions and Innovations to Overcome Chronic Kidney Disease (Can-SOLVE CKD) Strategic Plan, particularly “Theme 1: Identify kidney disease earlier and support those who are at highest risk of negative outcomes, including Priority 1.1: How can we identify those with or at risk for CKD earlier?,” with the goal of transforming kidney health care for Indigenous Peoples in Canada.^12^ Developed within the framework of Canada’s largest patient-oriented kidney research network, the program was designed to implement the Kidney Check national protocol—a standardized, community-based approach to screening for CKD, diabetes, hypertension, and associated risk factors.^9^

Kidney Check reflects a strategic response to long-standing calls for equitable, accessible, and culturally relevant health services in Indigenous communities. It was intentionally built in partnership with Indigenous governance structures and guided by Indigenous values, data sovereignty principles, and reciprocal accountability. Its success can be traced to two foundations: the First Nations Community-Based Screening to Improve Kidney Health and Prevent Dialysis (FINISHED) program,^13,14^ which served as the crucial proof-of-concept pilot, and the Can-SOLVE CKD Network,^15^ which provided the national strategic framework and resources necessary for expansion. Understanding this lineage is essential to appreciating Kidney Check as a model for how to effectively translate a successful local intervention into a sustainable, multi-provincial program.

The program is explicitly based on the FINISHED program,^13,14^ which was a groundbreaking three-year project (2013-2015) in Manitoba (MB) that demonstrated the feasibility and effectiveness of a mobile, POCT-based screening model for kidney disease in high-risk, underserved First Nations communities. Operating in 11 rural and remote communities, the FINISHED team proved that a mobile unit could successfully deploy POCT technology in challenging settings, effectively triaging high-risk patients into specialist care pathways with minimal delay. Most importantly, it established efficacy, finding that over a quarter of participants had some level of kidney disease. Among these, the vast majority (87%) are early-stage and potentially treatable—a critical window for intervention. The FINISHED project demonstrated feasibility, clinical impact, community acceptance, and cost-effectiveness, supporting larger-scale investment in early detection and prevention.^16-18^

While FINISHED provided the framework and implementation tools, the Can-SOLVE CKD Network provided the strategic architecture for national expansion. Can-SOLVE CKD is a patient-oriented research network established in 2016 as part of the Canadian Institutes of Health Research (CIHR) Strategy for Patient-Oriented Research (SPOR).^15^ In 2018, the Can-SOLVE CKD Network officially “took on the program,” recognizing the immense potential of the FINISHED model to help achieve its national mandate to ensure all Canadians receive the best recommended kidney care. The network became the strategic enabler for the program’s evolution into Kidney Check and its expansion beyond MB into British Columbia (BC) and Alberta, with key learnings informing programs in Ontario and Saskatchewan.^11^ It provided not only funding but also a national platform, a robust research infrastructure, and a philosophical framework perfectly aligned with the program’s grassroots ethos. The network’s core principles of patient partnership and Indigenous engagement created the ideal environment in which the community-led Kidney Check model could grow and thrive.^19^ Importantly, Indigenous patient partners with lived experienced with kidney disease, including patients, and their family members and caregivers, help guide implementation of the program in close collaboration with the research leads, ensuring the program aligns with the priorities and values of Indigenous people living with kidney disease:

As a First Nations woman and someone who benefitted from an early diagnosis of kidney disease at a routine physical when I was not sick, kidney screening is so important to improve the health of First Nations people to mitigate the risk of kidney failure, and its impact on our healthcare system and our communities. In Kidney Check, patient partners—people with lived experience of kidney disease, like myself—have played a vital role in ensuring the program’s success by helping make it more meaningful and relevant for the people it aims to serve.—Cathy Woods, Kidney Check Patient Partner

## Provincial Sources of Information, Methods, and Key Findings

The Kidney Check program is grounded in a model of co-creation and authentic partnership, guided by Indigenous methodologies and governance frameworks.^10^ This approach respects community autonomy and sovereignty, ensuring that each community determines how the program is implemented locally. Rather than imposing a standardized model, Kidney Check engages each community as an active partner in designing and delivering care. This approach is essential to building trust in communities where it has been eroded by historical and ongoing colonial experiences. Indigenous methodologies are central to the program, including a Two-Eyed Seeing approach that integrates Western biomedical and Indigenous ways of knowing.^20^ Governance follows the Ownership, Control, Access, Possession (OCAP) principles,^21^ ensuring community control of data.

**Figure 1.**
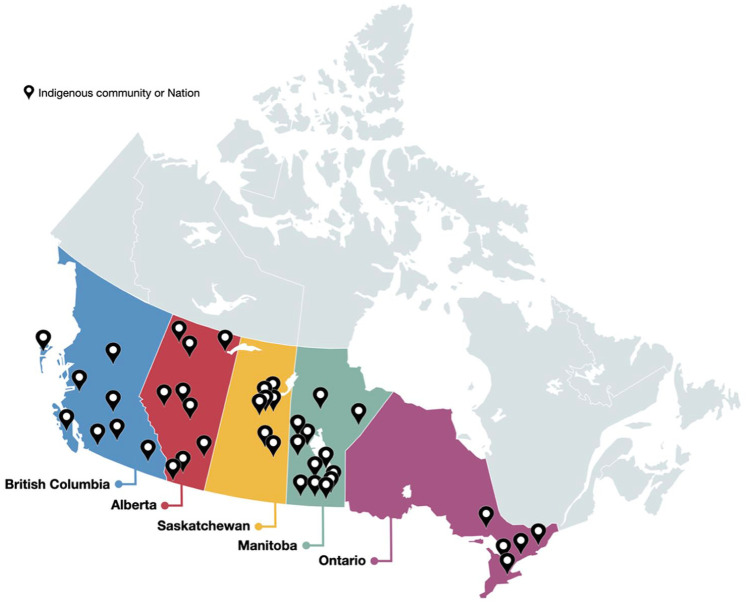
Distribution of communities participating in the Kidney Health Check (KHC) program across five provinces in Canada: Alberta, British Columbia, Manitoba, Ontario, and Saskatchewan.

**Figure 2.**
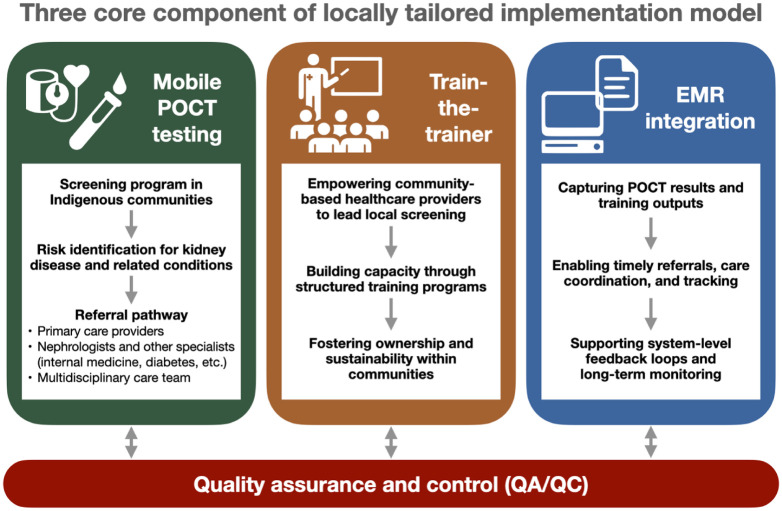
Core components of locally tailored implementation: mobile point-of-care testing for screening and risk identification in rural/remote communities, a train-the-trainer model to build local capacity, and EMR integration to support documentation, referrals, and coordinated care. EMR, electronic medical record; POCT, point-of-care testing; QA/QC, quality assurance and quality control.

The Kidney Check program is currently implemented across 3 provinces, and its methods have also informed CKD risk screening initiatives in two provinces in Canada (Figure 1), with each jurisdiction adapting the core model to reflect its specific community priorities, health systems, and logistical realities. Implementation is tailored to the local context and built on three core components (Figure 2). First, a mobile POCT model brings screening directly to rural and remote communities, addressing access and equity gaps. Second, a train-the-trainer approach builds local capacity by equipping community health care providers to lead screening efforts. All POCT operators undergo initial training and annual competency assessments to ensure quality and consistency. Third, the program integrates with health systems using electronic medical records (EMRs), facilitating documentation, referral management, and care coordination via clearly defined pathways to specialist care. These efforts are supported by tools such as the Can-SOLVE CKD Network’s “Pathway to Implementation Guide”^22^ and governed by standardized operating procedures led by Shared Health Diagnostic Services (MB), aligned with national accreditation standards.^22^

This methodology directly addresses several foundational pillars of health-system transformation. Cultural competency and patient partnership are strengthened through collaboration with Tribal Councils, Elders/Knowledge Keepers, and federal agencies such as Indigenous Services Canada. Sustainability and scalability are achieved by fostering community ownership, building local capacity, and leveraging national collaboration through the Can-SOLVE CKD Network’s CIHR funding.

The following section highlights how each province adapted the core model to meet local needs, priorities, and health care contexts.

## Alberta

### Methods

#### Organizational Structures Supporting Implementation

The Kidney Check program was implemented in AB beginning in 2018 through a collaboration between Indigenous communities, Alberta Health Services (AHS), and academic partners. The initiative was supported by a three-tiered organizational structure. The Leadership Team, composed of members from the University of Alberta, Can-SOLVE CKD Network, and an Elder from the Blackfoot Confederacy, provided oversight, secured funding, set protocol standards, and ensured cultural guidance. The Central Coordination Team, led by the AHS Diabetes, Obesity and Nutrition (DON) Strategic Clinical Network (SCN), acted as a liaison between teams, coordinated training and logistics, and supported ongoing implementation. Community Care Teams—comprising the Blood Tribe Department of Health (BTDH), Kainai Diabetes Team, Aakom-Kiyii Health Services (AKHS), and OKAKI’s, an Indigenous-led kidney health initiative, through its Community Diabetes Care Team—led onsite screening and community engagement. Support was also provided by the Chinook Primary Care Network (CPCN) and AHS South Zone Alberta Healthy Living Program (AHS SZ AHLP).

#### Implementation Process and Community Engagement

Initial collaboration between AHS and BTDH began in 2018, and local health care providers were trained. While screening was scheduled to launch in 2020, it was delayed by the COVID-19 pandemic. Engagement with Piikani Health and AKHS began in late 2021, and implementation planning resumed for both communities in 2022. Screening officially launched in March 2023 (BTDH) and April 2023 (AKHS) after a “train-the-trainer” session for local providers. A collaborative planning session in May 2024 brought together both communities and health care partners to discuss successes and challenges, reflect on progress, and plan next steps. These partners included staff from the OKAKI Community Diabetes Care Program, AHS SZ AHLP, and CPCN. That summer, the OKAKI team extended mobile screening to remote northern Alberta communities, including Fort Chipewyan, Montana, Fort Vermillion, High Level, and Alexis First Nations Communities.

#### Implementation Support Tools

**Figure 3.**
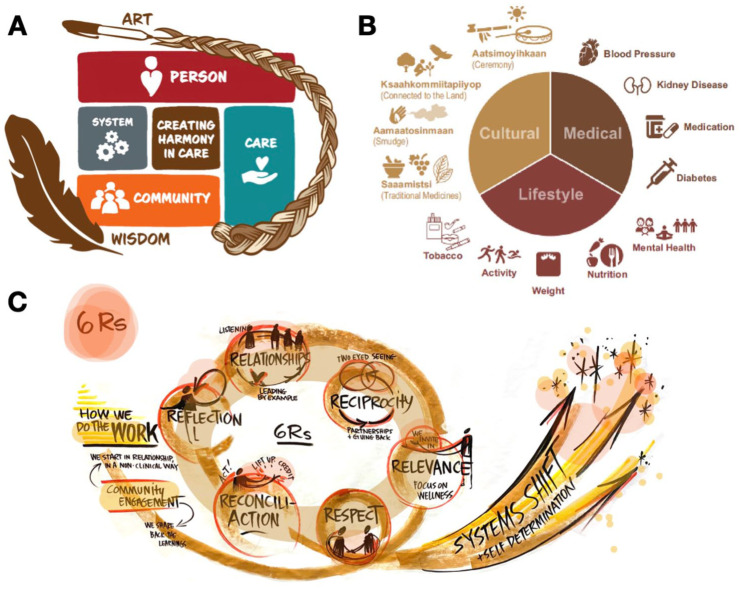
Frameworks guiding Kidney Health Check (KHC) implementation across provinces. Provincial adaptations of the KHC program are grounded in culturally safe, community-led approaches. (A) AB’s Creating Harmony in Care framework emphasizes person-, care-, community-, and system-level relationships, co-designed with Indigenous partners to foster trust and cultural safety. (B) The Wellness Model integrates medical, lifestyle, and cultural dimensions of health, reflecting Indigenous concepts of holistic well-being. (C) The six Rs of Indigenous Community Engagement—Respect, Relevance, Reciprocity, Relationships, Reflection, and ReconciliACTION—guide meaningful, sustained partnership. *Source.* *Panels (A) and (B) reprinted with permission from Alberta Health Services; panel (C) reprinted with permission from the British Columbia Renal Agency.*

Implementation in AB was guided by the Creating Harmony in Care framework,^23^ developed by AHS DON SCN in co-design with the local Indigenous health care providers, community wellness champions, and Elders (Figure 3A) and supported by an Indigenous Primary Health Care Policy Research Network (IPHCRH) seed grant.^24^ The model considers four key dimensions: person, community, care, and system. Fundamentally, that means this model is focused on addressing personal care, needs, and preferences, to help each person live a healthy life with, or at risk of, chronic disease. Participants were invited to share their experiences and feedback during and after screening visits, which helped refine processes and ensure that the program remained responsive to community priorities and values. Applying the Creating Harmony in Care model to Kidney Check meant building on existing community wisdom and resources (community) while embedding traditional and western evidence-based care, practices, and beliefs about chronic care into Kidney Check (care). Determinants of health, such as addressing access to care, transportation challenges, and food security, have been central in communication conversations related to helping people live a health life (system).

#### Capacity Building and Sustainability

The central coordination team provided an initial “Train the Trainer” Day in January 2023 for two communities. This was a collaborative day that focused on building capacity in the community and supporting peer-to-peer education. Training was provided by a registered nurse and project planner to the local health care providers, nurses, dietitians, and community liaison workers. Training materials included presentation slides and equipment manual with the POCT quality assurance and control (QA/QC) protocols. Content covered the overall screening process, participant flow, equipment uses, hands-on training, and the referral and follow-up pathway. The session also included an open discussion to plan the initial screening day, providing an opportunity to identify potential barriers and facilitators to support successful implementation. Initial screening events were supported by the Central Coordination Team to ensure QA/QC and peer-to-peer learning. Using Shared Health’s national protocols, local teams now manage equipment maintenance and can train new staff, fostering long-term sustainability.

#### Care Pathway—Alberta’s Wellness Model

In AB, Kidney Check focuses on screening people at risk of diabetes, hypertension, kidney disease, obesity, and diabetes-related eye and foot complications. Beyond screening, the program also addresses key risk factors through support for medical management, appropriate medication use, and promotion of healthy lifestyle practices. While only a subset of participants require referral to primary or specialty care, all individuals receive personalized coaching and resources to support disease prevention and health promotion.

To ensure comprehensive care pathway, the “Wellness Model” (Figure 3B) was co-created in alignment with Creating Harmony in Care^23^ and in collaboration with the project team and champions from Piikani’s Aakom-Kiyii Health Services team. This model focuses on providing people who are screened with access to:

- Medical supports such a chronic disease education (eg, diabetes, kidney, hypertension), medication management, and clinical follow-up.
- Lifestyle supports such as physical activity, exercise, nutrition, cooking, weight management, emotional and stress management, and appropriate tobacco use.
- Cultural supports, such as smudges, ceremonies, land-based education, and traditional medicines and practices.

#### Preliminary Evaluation of the Program to Guide Scale-up and Sustainability

To evaluate the implementation of Kidney Check in AB, we applied the Consolidated Framework for Implementation Research (CFIR)^25^ and the Working in Good Ways framework.^26^ The CFIR is an evidence-based tool used to assess contextual factors influencing implementation, organized into five domains: the intervention, outer setting, inner setting, individuals involved, and the overall implementation process.^25^ The Working in Good Ways framework, developed by the University of Manitoba through comprehensive consultations, provides practical strategies for respectful and effective community engagement with Indigenous partners throughout all stages of implementation.^26^

### Key Findings

#### Process Measures

A total of 30 health care providers have been trained through the Kidney Check. In 2023, 21 providers were trained (14 from AKHS and seven from BTDH) and nine in 2024 (seven from BTDH and two from OKAKI). In 2024, an additional nine providers were trained— seven from BTDH and two from OKAKI. Trainees included registered nurses, licensed practical nurses, nurse practitioners, registered dietitians, community health representatives, and kinesiologists.

Using qualitative descriptive methods, people involved in the implementation of Kidney Check were interviewed to identify key lessons and insights. Participants represented various roles, including program leadership, facilitation, and frontline delivery. Between July and August 2023, a total of 21 individuals participated in the evaluation across five focus groups and two individual-structured interviews. Thirteen participants were directly involved in delivering the program within two communities (Blood Tribe and Piikani), five supported program facilitation and coordination (eg, material development, equipment training), and three held leadership roles in program design and oversight.

#### Barriers to Implementation

Several challenges were encountered during the Kidney Check implementation in AB. Travel logistics and equipment transport made it difficult to deliver screening in remote locations, particularly given the need for environmental control. Reagents had short expiration periods, and difficulties in predicting supply needs led to shortages. Equipment QA/QC protocols were time-intensive and, at times, disrupted clinical workflows during screening events.

Other operational barriers included long participant appointment times (often up to one hour) and clinic hours limited to weekdays, both of which restricted accessibility for some community members. The program’s name also led to some confusion about its scope, with individuals perceiving it as solely focused on kidney health rather than a broader wellness initiative.

#### Facilitators

Key facilitators included early and sustained engagement with community leaders, elders, and health care providers, which helped build trust and ensure the program was culturally aligned and responsive. Strong communication channels, including the appointment of a dedicated program liaison, supported efficient coordination and rapid problem-solving.

The train-the-trainer approach and integration of Kidney Check into existing community health services increased local provider capacity and supported long-term sustainability. Flexibility in program delivery allowed customization to meet local needs. Community-based promotion, such as leveraging health fairs and social events, enhanced awareness and participation. Finally, the use of a multidisciplinary team, including dietitians, nurses, and social workers offering in-person health coaching, helped reinforce a holistic, wellness-focused approach to care. Overall, the program has been very well received, as can be seen by participant and provider testimonials in Table 1.

**Table 1.** Testimonials From Kidney Check Participants and Providers.

Program participant testimonials
*“I felt comfortable coming in, the staff was so welcoming and friendly.”*
*“Very knowledgeable, kind and caring staff. It is located close to home.”*
*“Good information was provided which will be very helpful for my well being.”*
*“Now I know and I can go from here to take care of myself.”*
*“I like that there is transportation available.”*
—*BTDH Kidney Check participants-patients*
Program provider testimonials
*“I think the success of our co-design was that we included more than healthcare providers in the design. We included, especially the Elders, the community champions, etc.”* —*Central Coordination team member*
*“Kidney Check creates a very nice opportunity to speak to community members about nutrition and health risk factors. And community members [attend] that might not necessarily come to a screening event or might not come and see us on a one-on-one basis. So, it’s a nice opportunity to catch people that might not normally come in for an appointment. And then to create follow up after that as well, which is really nice.”* —*BTDH health care provider*
*“I think it has to be at tailored and customized to what the community wants . . . the risk of these mobile clinics is . . . parachuting in and out. It’s not building capacity within the community. It’s not a sustainable model necessarily . . . I think working with the community and providing the service and the resources within the community [is] far superior model.”* —*Leadership team member*
*“What made Kidney Check successful is the commitment and willingness of the team to really, you know, have those conversations and gain that depth of understanding of what the possible journey will look like. Understanding a little bit more of what traditional medicine and wellness is, and a deeper depth of it, and also gaining a relationship where trust is built.”* —*Central Coordination team member*

## British Columbia

### Methods

#### Organizational Structures Supporting Implementation

The Kidney Check in BC was built on a foundation of long-term relationship-building, beginning in 2016 with the First Nations Health Authority (FNHA). The goal was to establish a collaborative partnership to deliver culturally safe kidney screening services for First Nations communities across the province. Following extensive engagement, a formal Expression of Interest (EOI) process was launched, inviting communities to participate. A total of 28 EOIs were received from communities across BC’s five health regions and the FNHA.

#### Implementation Process and Community Engagement

To ensure transparency and readiness, a community capacity assessment tool was developed to evaluate applicants’ uses a flexible, mixed-model ability to support and sustain screening activities. A formal selection committee—comprised of Indigenous patient partners, FNHA, First Nations Health Directors Association (FNHDA), Can-SOLVE CKD leadership, and Knowledge Keepers—was formed to guide site selection. This process culminated in the signing of a Memorandum of Understanding between Can-SOLVE CKD and FNHA, clearly outlining shared roles and responsibilities for delivering Kidney Check in BC.

Screening began in October 2019 with the Kyuquot First Nation on Vancouver Island in partnership with the Nuu-chah-nulth Tribal Council. Since then, implementation has expanded to more than 17 First Nations communities across Fraser Health, Interior Health, Vancouver Coastal Health, and Island Health regions. The program aims to screen 20% to 30% of eligible individuals in each participating community, reaching approximately 1000 participants during the initial implementation phase.

#### Implementation Support Tools

BC uses a flexible, mixed-model approach tailored to community needs. This includes three models: (1) a traveling screening team, (2) a community-based screening team, and (3) a hybrid of both. Each community leads its own screening initiative, supported by Can-SOLVE CKD and FNHA, and the approach remains community-led and capacity-driven. Ongoing engagement activities include lunch-and-learn sessions, community visits, and presentations at regional health caucuses, with further expansion and scale-up underway.

The BC Kidney Check program is grounded in Indigenous methodologies and governance, prioritizing OCAP principles and trauma-informed care. The Kidney Check coordinator has completed certified training in Indigenous and trauma-informed coaching. The program is guided by the six Rs of Indigenous Community Engagement—Respect, Relevance, Reciprocity, Relationships, Reflection, and ReconciliACTION—a framework developed by Can-SOLVE CKD’s Indigenous Initiatives team to ensure meaningful, ethical, and sustained community partnership (Figure 3C).^27^ Barriers and facilitators were identified through ongoing engagement and feedback from participating communities and the project team during implementation.

### Key Findings

#### Process Measures

As of July 2024, a total of 718 individuals have been screened through the program across First Nations communities. Over 50 screening events have been conducted, supported by more than 25 locally trained providers and five nurses trained for mobile screening activities.

#### Barriers to Implementation

Several barriers have impacted implementation across BC communities. Logistical challenges include travel to remote locations and securing accommodations, especially during tourist seasons. Transporting equipment and maintaining temperature-sensitive supplies is complex and costly, and ensuring consistent QA/QC across sites is resource-intensive. High costs of QC materials and short shelf lives add to the challenge.

Limited nursing capacity within communities and difficulty recruiting external agency nurses have also posed barriers. In addition, competing priorities within communities—such as emergencies, climate events, or community grief—can delay or deprioritize screening activities. Broader structural challenges include long-term sustainability, community engagement during program delivery, and ongoing financial constraints.

#### Facilitators

Implementation has been strengthened by grounding the program in Indigenous research methodologies that prioritize relationality, local knowledge systems, and wellness-focused engagement. Continuous community engagement is central, beginning with identifying Indigenous patient partners, aligning visits with community guidance, sharing meals, and receiving formal welcomes from Elders or Knowledge Keepers.

Strong partnerships with provincial and Indigenous health organizations—including FNHA, FNHDA, and the First Nations Health Council—have supported implementation, as well as a dedicated project team, including a project manager and registered or licensed practical nurse, who lead operations. A comprehensive training program emphasizes cultural safety, trauma-informed care, and community-based practice.

## Manitoba

### Methods

#### Organizational Structures Supporting Implementation

The Manitoba Kidney Check program builds on the foundation of the earlier FINISHED research initiative (2012-2015),^13^ which was developed through partnerships with the First Nations Social Secretariat of Manitoba (FNHSSM) and Shared Health Services Manitoba—Manitoba’s centralized health authority. Together, they developed a screen-triage-treat framework for individuals aged ≥10 years. These foundational partnerships continued through the Kidney Check phase, with Shared Health supporting equipment procurement, quality assurance, maintenance, and standard operating procedures (SOPs), and FNHSSM serving as a key implementation and data stewardship partner. The program later expanded through the CIHR SPOR–funded Can-SOLVE CKD Network, which enabled broader implementation across provinces. A new partnership was also established with Keewatinohk Inniniw Minoayawin Inc (KIM), whose mandate is to close the care loop by addressing gaps in access to primary care. The KIM supports referrals for adults without a primary care provider via a physician assistant, and for children via an itinerant pediatrician. Individuals identified as high-risk are referred to endocrinology or nephrology care as needed.

#### Implementation Process and Community Engagement

**Figure 4.**
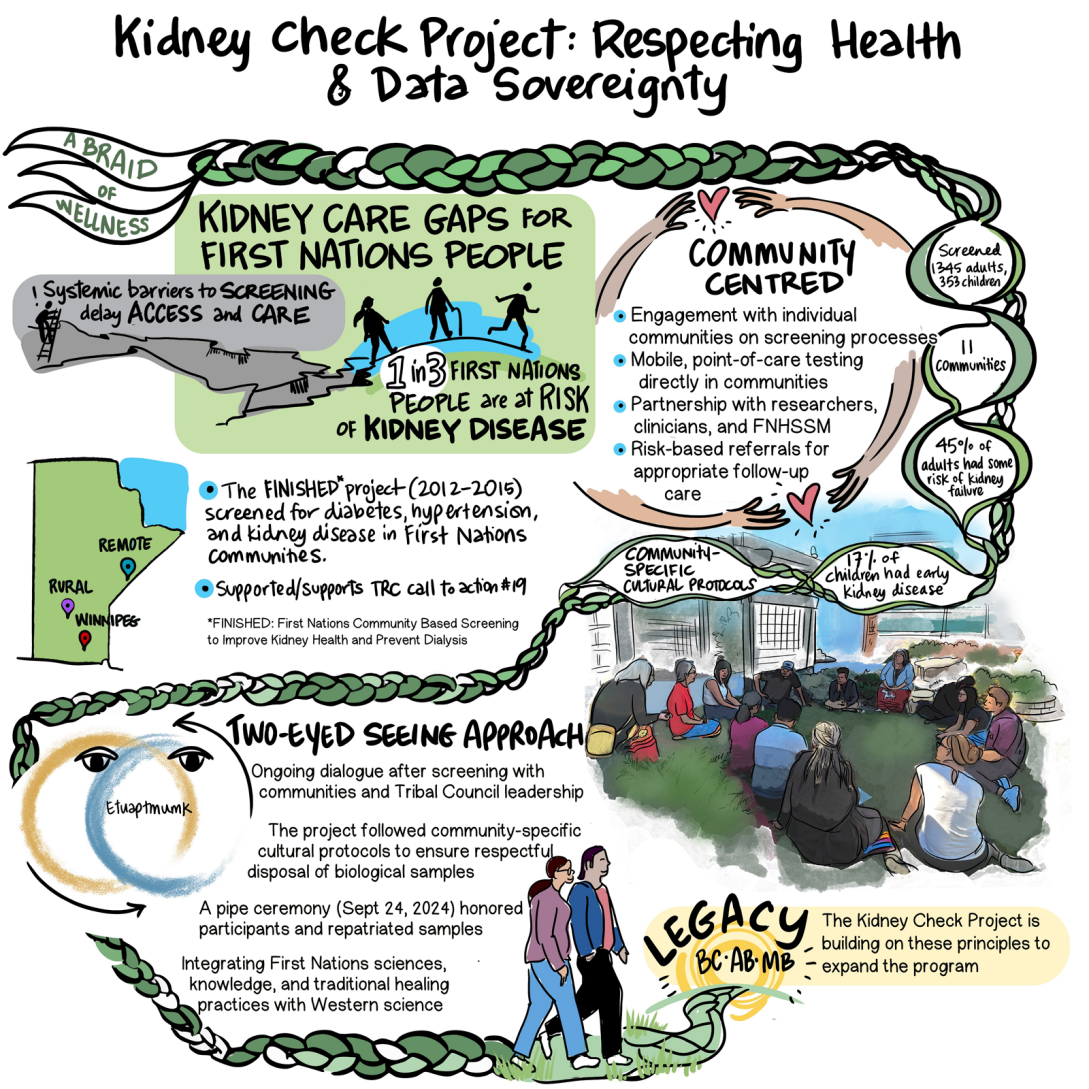
Kidney Check project: respecting health and data sovereignty through community-centered, culturally safe approach: an overview of the Kidney Check program in Manitoba, grounded in Indigenous-led approaches, cultural safety, and data sovereignty. *Source. Reprinted with permission from Shared Health Services Manitoba* (AB, Alberta; BC, British Columbia; FNHSSM, First Nations Health and Social Secretariat of Manitoba; MB, Manitoba). *Note*. The project builds on the FINISHED initiative and uses a Two-Eyed Seeing approach to deliver mobile, community-centered screening, guided by local protocols and partnerships. Ongoing community dialogue and integration of First Nations and Western knowledge systems ensure culturally safe and sustainable care delivery. This foundation supports program expansion across provinces.

Program implementation is tailored to each community and grounded in relationship-building and cultural safety. Beginning in 2018, a patient partner panel was formed to guide program branding, education materials, community selection, and operations. These partners ensured the program was community-centered and wellness-focused. The FNHSSM team travels to rural and remote communities to conduct multi-day screening events and host pop-up events at urban Indigenous organizations. Each event includes mobile POCT, personalized education based on real-time results, and facilitated referrals to appropriate care (Figure 4). A custom-built app supports onsite result interpretation and individualized health education.

#### Implementation Support Tools

Screening events are co-designed with local partners. A key goal of Kidney Check in MB has been to create the infrastructure to ensure long-term sustainability. Aiding this was the development of a screening framework, supported by robust SOPs, which could be adapted and applied to regional and community-based systems. For example, SOPs were adapted by the Population Health and Wellness Team from the Prairie Mountain Health Region, who conducted screening activities as part of a mobile public health screening program in Southwestern Manitoba from 2023 to 2024. This continued support reflects several factors unique to Manitoba, including the strong implementation infrastructure established through the FINISHED project, long-standing partnerships with FNHSSM and Shared Health Services Manitoba, and clear community demand for chronic disease screening embedded within routine care rather than as a time-limited research initiative. As Phase II grant funding ends for the Can-SOLVE CKD Network, the legacy of the Kidney Check in Manitoba will shine through its established, evidence-based infrastructure for sustainable screening of CKD, diabetes, and hypertension. This model can also be extended to other easily screened for and treatable conditions, such as hepatitis C or HIV. Long-term provincial public health funding will enable screener training and education, maintenance of POCT equipment, data management, and patient partner involvement, to screen all Manitoba First Nations communities on a rotating basis every 3 to 5 years with success metrics being the percentage of individuals ≥10 years of age screened and systematic uptake of disease-modifying medications post-screening by participants’ circle of care.

#### Capacity Building and Sustainability—“Train the Trainer” Approach

A train-the-trainer model was explored to support community-led screening. While promising in other provinces, pilots in Manitoba faced challenges due to staffing shortages and competing local priorities. To address this, Red River College, in partnership with Manitoba Keewatinowi Okimakanak Inc and KIM, launched a Diagnostic Support Worker Applied Certificate program in 2024. The program includes POCT micro-credentials and aims to build a trained Indigenous workforce capable of delivering in-community chronic disease screening over the long term.

### Key Findings

#### Process Measures

Between February 2023 and August 2025, the Kidney Check team traveled to 10 rural and remote Indigenous communities across Manitoba and held screening pop-up events at urban Indigenous and inner-city service organizations, cumulatively screening 685 individuals aged ≥10 years.

#### Barriers to Implementation

While overall a successful initiative, the program was impacted by some significant barriers. The COVID-19 pandemic, which interrupted the program as it was beginning to ramp up screening activities, served as a major barrier since rural and remote communities completely shut down to prevent the spread of the virus and were cautious with reopening. In response, the Manitoba team pivoted to a mail-based screening spinoff—the Virtual Kidney Check and Follow-up project—which used administrative health databases to identify and send laboratory requisitions, kidney health education materials, and accompanying letters by mail to adults who had not received kidney testing in the past 2 years.^28^ More recently, wildfires in the summers of 2024 and 2025 restricted travel and limited engagement with northern communities, as many focused on supporting neighboring areas facing evacuations. Facilitators for sustainability, including models of screening where local staff are trained to conduct screening and provided with screening equipment, have been attempted in other provinces and would enable continuity of Kidney Check in situations where external teams cannot travel into communities. Pilots of this model in Manitoba have not shown to be efficient due to limited health care resources and competing priorities. However, in November 2024, Red River College, in collaboration with Manitoba Keewatinowi Okimakanak Inc and KIM, established a Diagnostic Support Worker Applied Certificate program with POCT micro-credentials, with the ultimate objective of training highly qualified personnel who will contribute to building capacity in Indigenous communities to support in-community chronic disease screening.

#### Facilitators

Important legacies of the program include the development of a customized app that enables real-time results calculation, age-specific risk-based patient education, and streamlined data management. Data are stored on a secure, Protected Health Information (PHIA)- and Health Insurance Portability and Accountability Act (HIPPA)-compliant Canadian cloud-based server. While FNHSSM acts as data stewards, ownership and control rest with individual Tribal Councils. Program success depends on community-specific adaptation. Early and ongoing engagement—including returning screening reports to leadership—has been vital to building trust and honoring the TRC Calls to Action. Flexibility in screening hours and locations, collaboration with schools and Jordan’s Principle^29^ organizations, and promotion via local radio, social media, and community events have all increased awareness and participation. Finally, continuous patient partner involvement—especially in program design and community engagement—has been essential to the initiative’s relevance and sustainability.

## Ontario

### Methods

#### Organizational Structures Supporting Implementation

Since 2016, the Ontario Renal Network (ORN), under Ontario Health, has partnered with regional renal programs and First Nations communities to deliver the CKD Risk Screening Program across Ontario. The program is locally led by Regional Renal Program teams in collaboration with Indigenous health services, ensuring each initiative is co-developed to reflect local needs, capacity, and resources. Currently, 5 Regional Renal Programs offer screening services tailored to the needs, capacity, and resources of each participating community.

#### Implementation Process and Community Engagement

The first screening was launched by Health Sciences North in Sudbury in partnership with Wikwemkoong Unceded Territory and the Naandwechige-Gamig Wikwemikong Health Centre. A locally hired Registered Practical Nurse led delivery and referral tracking, resulting in the screening of over 500 participants in the first year and timely referrals to primary and specialty care. The current focus is on delivering screening services on-reserve, with plans to expand to off-reserve First Nations populations in the future.

#### Implementation Support Tools

Program delivery models are flexible, with screening led by either Regional Renal Program teams or trained community health workers, depending on local preferences and capacity. The implementation model prioritizes seamless, local referral pathways. Screening includes point-of-care HbA1c and blood pressure testing, followed by risk stratification using the Kidney Failure Risk Equation (KFRE),^30^ supported by serum creatinine-based eGFR and urine albumin measurements obtained during screening. Participants at high risk are referred to nephrology care at the nearest Regional Renal Program, while others are referred to community-based or virtual services for diabetes and hypertension management. This approach minimizes travel burden and improves the likelihood of follow-up rates. Screening is embedded into existing community health initiatives wherever possible (eg, diabetes, oncology, or mental health programs) to maximize coordination and engagement.

#### Capacity Building and Sustainability

Community-based providers receive training and ongoing support from the Regional Renal Programs, ensuring consistent delivery and QA/QC. To strengthen implementation capacity across the province, the ORN has established the CKD Risk Screening Community of Practice—a collaborative network that enables Regional Renal Programs to share processes, tools, lessons learned, and implementation strategies. This peer-learning forum supports scale-up, encourages continuous improvement, and fosters sustainability across sites. The involvement of locally hired staff has been critical to strengthening community ownership, improving continuity of care, and supporting long-term program sustainability.

### Key Findings

Most current screening initiatives began or resumed in 2024. From April 2024 to March 2025, over 250 individuals were screened through the CKD Risk Screening Program. As activities are still in the early stages, screening volumes remain modest, but early engagement of Indigenous health partners and Regional Renal Programs has supported re-establishment of services and laid the groundwork for future scale-up.

## Saskatchewan

### Methods

#### Organizational Structures Supporting Implementation

The Kidney Check Program, Saskatchewan Health Authority (SHA), based in Saskatoon, was established in 2017 to serve the northern half of the province. The mandate was to promote kidney disease risk awareness, education, and screening among high-risk populations. The program operates under nephrologist leadership and is delivered by a dedicated team consisting of one nurse clinician and one registered dietitian/diabetes educator. Over the years, Kidney Check has established long-standing partnerships with Indigenous communities, the Kidney Foundation of Canada, older adult organizations, and post-secondary institutions. Kidney Check Saskatchewan is funded by the St. Paul’s Hospital Foundation in Saskatoon, with in-kind operational support provided by SHA.

#### Implementation Process and Community Engagement

The implementation of Kidney Check officially launched in September 2023, following five years of preparatory work and strong collaboration with Indigenous communities, which facilitated a smooth program rollout. Demand for Kidney Check has already exceeded current capacity, highlighting the need for future expansion. The program currently operates as a mobile screening model, serving seven Indigenous communities across northern and northeastern Saskatchewan.

#### Implementation Support Tools

The program adopted initial SOPs from the MB Kidney Check, and additional SOPs specific to POCT—including QA/QC protocols—were developed by SHA Laboratory Services. All screening data are securely entered into the SHA Kidney Health information system, which is accessible only to the SHA Kidney Health team. Participants’ primary care providers receive detailed reports with screening results and clinical recommendations, while each participating community is provided with semi-annual summary reports to support transparency and shared learning. Public kidney health education sessions are also offered upon request.

Screening events are typically organized in collaboration with local health care providers and are frequently integrated with other outreach programs, including screening for sexually transmitted and blood-borne infections, liver health, and immunization services. Kidney knowledge surveys and post-event evaluations are completed in partnership with communities to gather feedback and guide program improvement. Individuals identified as being at intermediate or high risk for kidney failure are referred to nephrologists in Saskatoon for further assessment and follow-up care.

#### Capacity Building and Sustainability

Although Kidney Check operates with a small core team, collaboration with local health providers during screening events supports knowledge exchange and skills development. When feasible, Kidney Check staff involve community providers directly in the screening process to build local capacity. Community feedback is consistently used to guide quality improvement and ensure cultural and contextual relevance. The program’s integration with existing outreach services and its reliance on local partnership networks have been key to its continued reach and sustainability.

### Key Findings

#### Process Measures

Since the launch of Kidney Check Saskatchewan in September 2023, a total of 230 participants have been screened across 22 screening events. Of these, seven participants were screened twice, and one participant was screened three times. Among all individuals screened, five were identified as having an intermediate risk of kidney failure, and one participant was classified as high risk. The most common risk factors among participants were commercial tobacco use (64%), hypertension (40%), and diabetes (34%).

#### Barriers to Implementation

Despite the overall success, several implementation challenges were encountered. Provincial regulations related to the operation of POCT, including equipment-specific training and the time required for quality assurance and control processes, posed logistical and resource burdens. Community-level challenges also affected program delivery, including the limited availability of health care staff due to existing workload demands and unexpected events such as forest fires and local tragedies. In addition, staffing limitations within the Kidney Check constrained the program’s capacity to meet the growing demand for screening services.

#### Facilitators

Several key facilitators supported successful implementation. The long-standing, trust-based relationships with Indigenous communities and organizations played a central role in enabling effective community engagement and fostering program acceptance. Participants expressed deep appreciation for receiving immediate results and in-person health coaching, which enhanced understanding and promoted empowerment. The involvement of local health care providers, including those from primary care, further strengthened the program’s integration into existing services. Ultimately, the success has been closely tied to its flexibility and commitment to adapting the program to each community’s unique needs while upholding principles of respect, safety, dignity, and empathy.

#### Patient and Patient Partner Voice/Testimonials

We learned so much today, and you didn’t skip anything, you listen to us and take the time to explain. We need that!” (participants-patients)

Finally, someone who can tell me things about my diabetes that all the doctors and NPs over the years never did because they have no time. I never understood till today how diabetes affects my kidneys. (participants-patients)

I felt safe asking questions. Awesome! (participants-patients)

## Limitations

Despite its achievements, the Kidney Check has several limitations. First, the variation in infrastructure, staffing, and data systems across jurisdictions presents challenges to harmonizing implementation and evaluation nationally. However, what should be harmonized are the overarching principles and core approach, whereas the operational details must remain adaptable to local circumstances, community priorities, and existing health-system structures. While provinces adapted core protocols, differences in EMR integration, data accessibility, and referral pathways can limit cross-site comparability. Moreover, because several provinces are still in early stages of implementation, meaningful comparison of success factors and barriers—including trend analyses and economic evaluation—will be more feasible once the implementation have matured. Second, the reach of the program is still constrained by human resources—many regions continue to operate with small teams, limiting the frequency and geographic breadth of screening events. Third, although qualitative feedback from participants has been overwhelmingly positive, formal long-term outcome evaluations—including impact on disease progression, care engagement, cardiovascular events, and mortality—are ongoing and not yet available. Fourth, as the program continues to expand, maintaining cultural safety, community ownership, and fidelity to local protocols will require careful balance to avoid unintended centralization or loss of community control. Finally, the absence of a clearly defined long-term funding model and governance structure raised concerns about program sustainability. Encouragingly, several provinces are working toward embedding Kidney Check within existing Indigenous-led and provincial care structures, which supports continuation, but stable long-term funding beyond the Can-SOLVE CKD Network remains essential.

## Implications

The Kidney Check program stands as a landmark Canadian innovation in public health and health equity. Its success lies in the intentional integration of Indigenous worldviews, community wisdom, patient partnership, and clinical technology.^10^ This co-developed model of care addresses a long-standing gap in early detection and chronic disease prevention among Indigenous populations by embedding cultural safety, community self-determination, and clinical excellence throughout all stages of implementation.

### Key Learnings

The success of the Kidney Check is rooted in a set of foundational principles and implementation strategies that offer a clear blueprint for improving health equity and chronic disease prevention among Indigenous populations. These principles underpin Kidney Check nationally, although each province applies them in ways that reflect its local context, partnerships, and health-system structures.

#### Authentic Partnership and Community Self-Determination

Strong engagement, trust, and co-design with communities have been foundational to success across all provinces. Each nation led its own implementation, ensuring that the program aligned with local priorities, capacities, and governance structures. This commitment to community self-determination was upheld throughout planning, delivery, and follow-up.

#### Cultural Safety as a Clinical Imperative

Kidney Check centered cultural safety not as a supplementary component but as a clinical necessity. Frameworks such as Creating Harmony in Care,^23^ Two-Eyed Seeing,^20,31^ RE-AIM (Reach, Effectiveness, Adoption, Implementation, and Maintenance),^32^ and adherence to OCAP/Ownership, Control, Access, Stewardship (OCAS) principles^21^ guided every aspect of implementation. These Indigenous-led approaches ensured relational accountability, respect for data sovereignty, and alignment with local values and worldviews.

#### Capacity-Building and Local Empowerment

Train-the-trainer models were used to build local capacity, empowering community health care providers and supporting long-term sustainability. Public education, culturally grounded coaching, and collaboration with local health systems further reinforced the model’s effectiveness and adaptability.

#### Technology to Overcome Systemic Barriers

Mobile POCT compressed the care cascade, enabling immediate clinical assessments and timely intervention in remote communities. This decentralized approach addressed long-standing barriers related to distance, logistics, and service availability.

#### Data Translation Into Actionable Knowledge

The KFRE^30^ helped transform complex clinical data into meaningful and comprehensible information. This enabled shared decision-making and enhanced health literacy, allowing participants to engage actively in their care.

#### Patient-Centered Design and Culturally Grounded Approaches

The program was intentionally designed around the individual’s health journey—acknowledging readiness for change and complex lived experiences. In-person coaching and culturally appropriate follow-up supported empowerment, trust, and continuity of care.

#### Flexible, Iterative, and Scalable Implementation

Implementation models varied—ranging from mobile outreach teams to community-delivered services—but all upheld principles of co-creation and adaptability. Centralized coordination remained essential for training, equipment maintenance, and quality assurance. The iterative pilot-to-scale pathway, initiated with the MB-based FINISHED project,^13^ ensured continuous learning and context-specific scaling.

Participants across jurisdictions consistently emphasized feeling respected, heard, and supported—highlighting that cultural alignment, personalized education, and relational care are key to both acceptability and impact.^33^ Together, these factors demonstrate that culturally safe, community-led chronic disease screening is not only possible but powerful.

### Equity Considerations

Equity remains at the core of the Kidney Check program. It upholds data sovereignty, patient partnership, and Indigenous self-determination, while ensuring system integration through EMRs and structured referral pathways. The program prioritizes community-defined success metrics, cultural safety, and accessible, decentralized service delivery. Through collaborative partnerships with Tribal Councils, Indigenous Services Canada, and local health care systems, Kidney Check demonstrates how health care equity can be advanced by aligning clinical goals with Indigenous values of respect, relevance, reciprocity, and reconciliation.

### Future Directions

Kidney Check offers a powerful, replicable model for chronic disease prevention among underserved populations. Future directions include expanding coverage to all Indigenous communities across Canada, including off-reserve and urban populations. A long-term goal is to transition toward fully community-led implementation supported by sustained provincial and federal funding. In addition, embedding Kidney Check within broader health initiatives—such as diabetes, liver health, and mental wellness—offers opportunities for integrated, efficient service delivery. However, integration must preserve Kidney Check’s core principles to avoid diluting the strengths of the model.

### Policy Implications and Recommendations

The evidence presented in this report supports several key recommendations for interest holders across the Canadian health landscape:

- **For funding bodies:** Funding priorities should be reoriented to explicitly value and reward programs that demonstrate deep community partnership and patient-oriented design. Granting processes should recognize that investments in the “soft infrastructure” of relationship-building, community engagement, and cultural safety are essential for program effectiveness and yield significant long-term returns.
- **For provincial/territorial health systems:** Health authorities should invest in mobile health infrastructure and POCT capabilities to decentralize diagnostic services, particularly for rural, remote, and other underserved populations. System leaders should explore how the core principles of the Kidney Check model can be adapted to other chronic disease strategies, such as those for diabetes, hypertension, and cardiovascular disease.
- **For researchers and academic institutions:** There is a need for continued, rigorous evaluation of programs like Kidney Check, including further economic analyses to quantify their long-term cost-effectiveness and value for money. Research should prioritize mixed-method approaches that capture not only clinical outcomes but also the rich qualitative data of the patient and community experience, using implementation science methods to better understand “why” the model works.

## Conclusion

Kidney Check offers a powerful vision for the future of health care in Canada. It is a testament to the fact that the most complex challenges in health equity can be addressed when the wisdom of communities is centered, when patients are treated as true partners, and when technology is thoughtfully deployed in the service of people. It is a model that proves it is possible to create a health care system that is not only more effective and efficient but also more equitable.
